# CLImAT-HET: detecting subclonal copy number alterations and loss of heterozygosity in heterogeneous tumor samples from whole-genome sequencing data

**DOI:** 10.1186/s12920-017-0255-4

**Published:** 2017-03-15

**Authors:** Zhenhua Yu, Ao Li, Minghui Wang

**Affiliations:** 1 School of Information Science and Technology, University of Science and Technology of China, Hefei, AH230027 China; 2 Centers for Biomedical Engineering, University of Science and Technology of China, Hefei, AH230027 China

**Keywords:** Copy number alteration, Loss of heterozygosity, Whole-genome sequencing, Intra-tumor heterogeneity, Hidden Markov model, Bayesian information criterion

## Abstract

**Background:**

Copy number alterations (CNA) and loss of heterozygosity (LOH) represent a large proportion of genetic structural variations of cancer genomes. These aberrations are continuously accumulated during the procedure of clonal evolution and patterned by phylogenetic branching. This invariably results in the emergence of multiple cell populations with distinct complement of mutational landscapes in tumor sample. With the advent of next-generation sequencing technology, inference of subclonal populations has become one of the focused interests in cancer-associated studies, and is usually based on the assessment of combinations of somatic single-nucleotide variations (SNV), CNA and LOH. However, cancer samples often have several inherent issues, such as contamination of normal stroma, tumor aneuploidy and intra-tumor heterogeneity. Addressing these critical issues is imperative for accurate profiling of clonal architecture.

**Methods:**

We present CLImAT-HET, a computational method designed for capturing clonal diversity in the CNA/LOH dimensions by taking into account the intra-tumor heterogeneity issue, in the case where a reference or matched normal sample is absent. The algorithm quantitatively represents the clonal identification problem using a factorial hidden Markov model, and takes an integrated analysis of read counts and allele frequency data. It is able to infer subclonal CNA and LOH events as well as the fraction of cells harboring each event.

**Results:**

The results on simulated datasets indicate that CLImAT-HET has high power to identify CNA/LOH segments, it achieves an average accuracy of 0.87. It can also accurately infer proportion of each clonal population with an overall Pearson correlation coefficient of 0.99 and a mean absolute error of 0.02. CLImAT-HET shows significant advantages when compared with other existing methods. Application of CLImAT-HET to 5 primary triple negative breast cancer samples demonstrates its ability to capture clonal diversity in the CAN/LOH dimensions. It detects two clonal populations in one sample, and three clonal populations in one other sample.

**Conclusions:**

CLImAT-HET, a novel algorithm is introduced to infer CNA/LOH segments from heterogeneous tumor samples. We demonstrate CLImAT-HET’s ability to accurately recover clonal compositions using tumor WGS data without a match normal sample.

**Electronic supplementary material:**

The online version of this article (doi:10.1186/s12920-017-0255-4) contains supplementary material, which is available to authorized users.

## Background

Cancer is a disease that develops through accumulation of various genetic variability [1]. The clonal principles of tumor progression states that once initialization of a single founder cell is activated, various factors associated with carcinogenesis permit the descendants of the founder cell to resist apoptosis and undergo clonal growth, accompanying with acquirement of genomic alterations through multiple rounds of clonal expansion, and ultimately to invade neighboring tissues and metastasize to distant organs [1–5]. A tumor is thus heterogeneous and a mixture of multiple cell populations, and each population is characterized by a distinct complement of genomic aberrations. Genomic aberrations consist of somatic mutations such as single-nucleotide variations (SNV), copy number alterations (CNA), loss of heterozygosity (LOH), and more complicated structure variations (SVs) including inversion, translocation and etc., of which CNA and LOH are two frequently observed features of cancer genomes, and accurate detection of these aberrations is a crucial step to identify cancer-causing genes [6, 7].

Cancer-associated studies have been greatly promoted by continuous advances in experimental technologies for landscape of cancer genomes [8–12]. With the advance of sequencing technologies, high-throughput DNA sequencing presents an unprecedented advantage in deconvolving intra-tumor heterogeneity and detecting subclonal aberrations compared with array-based technologies. Whole-genome sequencing (WGS) of tumor samples is now a generally adopted approach for comprehensive analysis of structural and nucleotide-level aberrations that underpin tumor progression [13]. However, analysis of WGS data is generally complicated by several issues. For example, tumor sample is usually impure and mixture of cancerous and normal cells [9, 14]. The fraction of cancerous cells is usually represented as tumor purity. Low tumor purity will significantly diminish sequencing-derived signals, making it complicated to distinguish between aberrant and normal regions. Another intractable issue associated with cancer genomes is aneuploidy, caused by deletions or duplications of genomic segments or entire chromosomes in cancer genomes, and the average copy number of cancer genome is usually unknown [15–17]. One other more complex issue is the intra-tumor heterogeneity that results from the ongoing subclonal evolution [18], and the underlying clonal architecture is usually unavailable when dealing with patients’ tumor samples.

**Fig. 1:**
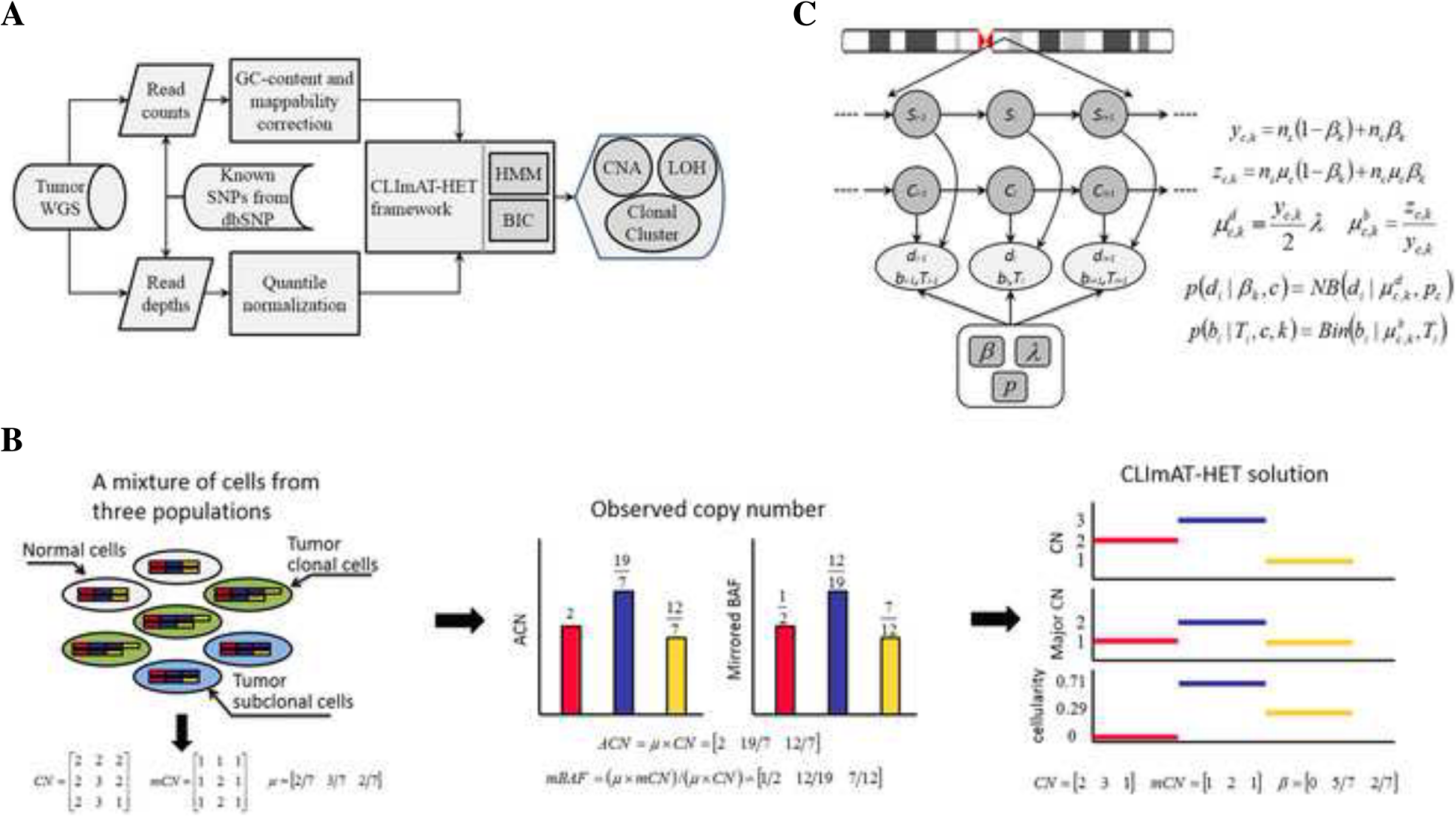
Overview of the CLImAT-HET statistical framework. **a** CLImAT-HET analysis workflow. 1) Read counts and read depths of known SNP positions are extracted from whole-genome sequencing data of tumor sample; 2) read counts signals are preprocessed to correct GC-content and mappability bias, quantile normalization of read depths is performed to eliminate allelic bias; 3) read counts and read depth signals are jointly analyzed using an integrated hidden Markov model, and the model complexity is iteratively evaluated for different number of clonal clusters using Bayesian information criterion; 4) and finally the clonal/subclonal CNA and LOH segments as well as the cellularity of each clonal cluster are inferred. **b** Representation of the intra-tumor heterogeneity and CLImAT-HET solution. The observed copy number signals are generated from three types of cell populations: normal cells, tumor cells with an amplification, and tumor cells with the amplification event and an additional deletion event. CLImAT-HET infers the total and major copy number as well as the corresponding cellularity of each event. **c** The factorial hidden Markov model adopted in CLImAT-HET. The hidden Markov model has two underlying Markov chains with one chain depicting aberration events and another delineating corresponding clonal clusters

In this study, we present CLImAT-HET, a novel algorithm based on the framework of CLImAT [19], for inferring subclonal CNA and LOH segments by taking into account the intra-tumor heterogeneity issue, in the case where a reference or matched normal sample is absent. The model jointly explores both read counts and allelic read depths at known SNPs across the whole genome from tumor WGS data (Fig. 1a), and quantitatively represents the copy number profiles of multiple subclonal populations, which is one novelty of CLImAT-HET. For each aberration event, we assume observed signals result from the joint effects of three distinct populations of cells: normal cells, tumor cells harboring the event and tumor cells without the aberration (Fig. 1b). The cellularity of CNA/LOH event is defined as the proportion of cells harboring the event. We further assume that multiple co-occurring events can be designated to one of a finite number of clonal populations. We adopt a factorial hidden Markov model (HMM) with two underlying Markov chains to delineate genomic aberrations and clonal clusters (Fig. 1c). In the factorial HMM, one Markov chain depicts genome aberrations and another represents the corresponding clonal clusters. Furthermore, the structure of the factorial HMM is automatically optimized by using an embedded model selection module based on Bayesian information criterion (BIC), which is another novelty of CLImAT-HET.

In contrast to related approaches in the literature, our method presents several distinct characteristics. APOLLOH [13] is designed to infer LOH from tumor sequence data, however it does not take into account the issue of tumor heterogeneity. PurBayes [20] uses somatic single-nucleotide variants derived from paired tumor-normal samples to estimate tumor cellularity and subclonality, and is only applicable to diploid tumor samples due to no integration of tumor ploidy and CNA information. THetA [21] is designed to infer cancer subclones by addressing a maximum likelihood mixture decomposition problem based on paired tumor-normal samples, but it cannot automatically determine the number of underlying subclonal populations. A recently developed method, TITAN [22], is designed to jointly call CNA and LOH segments from WGS data of tumor-normal paired samples. TITAN introduces a delicate factorial HMM to infer tumor subclones and performs an exhaustive search for the number of clonal populations up to 5 to find the optimal model structure. Our approach differs from TITAN in two aspects: 1) CLImAT-HET uses only single tumor sample and no reference or matched normal sample is required, and 2) CLImAT-HET learns the model structure under a BIC. Another method called OncoSNP-SEQ [23] accounts for intra-tumor heterogeneity by employing a non-clustering approach to model the distinct cell populations [22].

We perform a comprehensive evaluation of CLImAT-HET using simulated and real WGS data. By quantitative benchmarking on simulated datasets, we demonstrate that CLImAT-HET has the capacity to accurately infer cellularity of clonal clusters and subclonal CNA/LOH segments, and shows significant advantages when compared with existing approaches. We apply CLImAT-HET to 5 primary triple negative breast cancer (TNBC) samples to show its ability to identify clonal diversity in the CNA/LOH dimensions.

## Methods

### CLImAT-HET pipeline

The pipeline of CLImAT-HET is illustrated in Fig. 1a. The inputs to the model are extracted from WGS data using our previously published tool DFExtract [19]. Our analysis covers more than 2.6 million known SNPs along the whole genome. Copy number data of *N* SNPs is represented by read counts *d*_1:*N*_, meanwhile B allele frequency is represented by B-allele read depth *b*_1:*N*_ and total read depth *T*_1:*N*_. Following the procedures adopted in CLImAT [19], read counts are obtained by counting the reads within a 1000-bp window centered at each SNP, and further processed to correct GC-content and mappability bias. For B allele frequency, quantile normalization of read depths is automatically performed to eliminate allelic bias based on selection of optimal threshold by a grid search. CLImAT-HET then jointly analyze *d*_1:*N*_, *b*_1:*N*_ and *T*_1:*N*_ using an integrated HMM, and iteratively examine the model complexity using BIC for different number of clonal clusters, and finally output clonal/subclonal CNA and LOH segments as well as the cellularity of each clonal cluster.

### The statistical models in CLImAT-HET

It is intractable to precisely depict the genome-wide aberration status of tumors containing multiple subclones, therefore we adopt a simple assumption that the observed signals at a genomic locus are generated from underlying three types of cell populations: normal (non-tumor) cells, tumor cells with normal genotype, and tumor cells harboring the aberration event of interest (Fig. 1b). Thus, cell populations can be ultimately divided into two parts at a genomic locus: one with normal genotype and relative abundance of (1–*β*), and another harbors the aberration event with cellularity of *β*. To avoid extensive local parameters, we further assume all the aberration events can be clustered into a finite number of *K* groups, of which the *k*th group corresponds to the *k*th clonal population with respective cellularity of *β*_*k*_ (*β*_*1*_ *˂β*_*2*_ *˂…˂β*_*K*_), and tumor purity is equal to *β*_*K*_. Based on these assumptions, the model parameters can be effectively inferred by borrowing statistical approaches.

We define a set of states to depict copy number status of tumor genomes (Table S1, Additional file 1). Each state is represented by copy number, tumor genotypes derived from the normal genotypes by the deletion or duplication of alleles and aberration type. We only consider copy number status up to 7 copies, which is empirically based on the fact that most existing CNA-calling methods use this value as an upper bound [9, 19, 23]. Based on the emission models proposed in our previous study [19], we extend the conditional probabilities of read counts and B-allele read depth by introducing the contributions of multiple clonal populations. Given the aberration state *c* and the *k*th clonal cluster, we assume B-allele read depth is binomial distributed with the conditional probability defined as follows:

1 $$ p\left({b}_i\Big|{T}_i, c, k\right)={C}_{T_i}^{b_i}{\left({z}_{c, k}/{y}_{c, k}\right)}^{b_i}{\left(1-{z}_{c, k}/{y}_{c, k}\right)}^{T_i-{b}_i} $$

In formula (1), we only consider heterozygous genotypes in each aberration state. The mean of B allele copy number *z*_*c*,*k*_ and total copy number *y*_*c*,*k*_ are defined as:

2 $$ {z}_{c, k}={n}_s{\mu}_s\left(1-{\beta}_k\right)+{n}_c{\mu}_c{\beta}_k $$

3 $$ {y}_{c, k}={n}_s\left(1-{\beta}_k\right)+{n}_c{\beta}_k $$

where *n*_*s*_ and *u*_*s*_ deonte the copy number and expected B allele frequency (BAF) of normal cells respectively, *n*_*c*_ and *u*_*c*_ represent the copy number and BAF of tumor cells respectively. In addition, we assume read counts is negative binomial (NB) distributed with the conditional probability defined as follows:

4 $$ p\left({d}_i\Big|\lambda, {p}_c, c, k\right)=\frac{\varGamma \left({d}_i+{\lambda}_{c, k}\left(1-{p}_c\right)/{p}_c\right)}{\varGamma \left({d}_i+1\right)\varGamma \left({\lambda}_{c, k}\left(1-{p}_c\right)/{p}_c\right)}{\left(1-{p}_c\right)}^{\left({\lambda}_{c, k}\left(1-{p}_c\right)/{p}_c\right)}{p}_c^{d_i} $$

where *λ* is mean read counts associated with normal copy, and *λ*_*c*,*k*_ is formulated as:

5 $$ {\lambda}_{c, k}=\frac{y_{c, k}}{2}\lambda $$

The meanings of all other parameters involved in above formulas are the same as the ones described in CLImAT, and we do not go into detail here.

The conditional probabilities described above rely on two latent variables, aberration state *c* and clonal cluster *k*, therefore we implement CLImAT-HET as a factorial HMM with *C* × *K* hidden states by combining tumor genotypes and clonal clusters, where *C* is the number of aberration states defined in Table S1 and *K* is the number of clonal clusters. The HMM thus has two underlying Markov chains with one chain depicting aberration state sequence and another delineating corresponding clonal clusters (Fig. 1c). We employ expectation maximization (EM) algorithm [24] to learn the model parameters *θ* = (*π*, *A*, *β*, *λ*, *p*), where *π* is the initial state probability distribution, *A* is the state transition matrix, *β* is the cellularity of all clonal clusters, *λ* denotes the copy neutral read counts, and *p* refer to the success probability as a parameter of NB distributions. For the expectation step of EM algorithm, we calculate the expectation of the partial log-likelihood function of read counts and B-allele read depth respectively, and formulated as:

6 $$ E\left( L{L}_d\right)={\displaystyle \sum_{i=1}^N{\displaystyle \sum_{c=1}^C{\displaystyle \sum_{k=1}^K{\gamma}_{i, c, k} \log \left( p\left({d}_i\Big|\lambda, {p}_c, c, k\right)\right)}}} $$

7 $$ E\left( L{L}_b\right)={\displaystyle \sum_{i=1}^N{\displaystyle \sum_{c=1}^C{\displaystyle \sum_{k=1}^K{\gamma}_{i, c, k} \log \left( p\left({b}_i\Big|{T}_i, c, k\right)\right)}}} $$

where *γ*_*i,c,k*_ is the posterior probability of the *i*th SNP being in aberration state *c* and the *k*th clonal cluster, and is calculated using forward-backward algorithm [25]. For the maximization step, we use Newton-Raphson method [26] to update all model parameters until predefined convergence criterion is met. We define relative increment of the value of the log-likelihood function from iteration *n*-1 to *n* as follows:

8 $$ I n c=\frac{2\ast \left| L{L}_n- L{L}_{n-1}\right|}{\left| L{L}_n\right|+\left| L{L}_{n-1}\right|} $$

where *LL*_*n*_ is the value of the log-likelihood function in the *n*th iteration. If the value of *Inc* is less than a specific threshold (1 × 10^−4^), then the parameter updating procedure is stopped.

Given the number of clonal clusters *K*, the EM algorithm is implemented as follows: 1) start with initial parameters *θ* ^*0*^ = (*π* ^*0*^,*A* ^*0*^,*β* ^*0*^,*λ* ^*0*^,*p* ^*0*^) and calculates the joint-posterior probability *γ*_*i,c,k*_, 2) update *θ* ^*1*^ = (*π* ^*1*^,*A* ^*1*^,*β* ^*1*^,*λ* ^*1*^,*p* ^*1*^) using Newton–Raphson method, 3) repeat steps (1) and (2) until a specified number of iterations are reached or the convergence criterion is met. The converged parameters $\widehat{\theta}=\left(\widehat{\pi},\widehat{A},\widehat{\beta},\widehat{\lambda},\widehat{p}\right)$ in the last iteration of the training process will be output as the optimal estimators. Furthermore, we perform a grid search of the initial parameters *θ* ^*0*^ = (*π* ^*0*^,*A* ^*0*^,*β* ^*0*^,*λ* ^*0*^,*p* ^*0*^) to find the globally optimal solution.

As a vital parameter of CLImAT-HET model, the number of clonal clusters is determined using BIC. The BIC of a model is defined as follows:

9 $$ B I C=- \ln \widehat{L}+\frac{\alpha}{2} m \ln (N) $$

where $\widehat{L}$ is the maximized likelihood of the model, *α* > 0 is the regularizing term, *m* is the number of free parameters to be estimated and *N* is the number of SNPs. Our goal is to find an optimal value of *K* that leads to the model with the minimum value of BIC. A feasible solution is to perform an exhaustive search for possible values but it is practically intractable. Alternatively, CLImAT-HET starts with the initial assumption of tumor homogeneity (*K* = 1) and then iteratively increases clonal cluster number by one (*K* = *K* + 1) until the BIC value of the model no longer decreases. The optimal model is thus the one in the final iteration. For the computational convenience, we do not directly calculate the BIC of the model, but the difference of BIC between two adjacent models. Suppose that the number of clonal clusters is *n* in the *n*th iteration, and the BIC difference between the current model and the previous model is defined as follows:

10 $$ dBIC= BI{C}_n- BI{C}_{n-1}=-\Delta {\widehat{L}}_n+\frac{\alpha}{2}\Delta {m}_n \ln (N) $$

where $\Delta {\widehat{L}}_n$ and Δ*m*_*n*_ are the increment of the likelihood and the number of free parameters from model *n*-1 to model *n*, respectively. Δ*m*_*n*_ is measured by formula as follows:

11 $$ \Delta {m}_n=\left(2\left( n-1\right)+1\right){\left( C-1\right)}^2+2\left( C-1\right)+1 $$

The iteration is stopped once the BIC difference is greater than zero. After the model learning is finished, aberration information including copy number, tumor genotype, aberration type and clonal cluster for each SNP can be inferred from the hidden state with the maximum posterior probability. At the same time, segmentation of all SNPs based on hidden states is performed to output clonal/subclonal CNA and LOH segments.

Similar to the method described in CLImAT, we define a reliability score for the *i*th segment as follows:

12 $$ {S}_i= mean\left(\frac{p\left({b}_{i j}\Big|{T}_{i j}, c, k\right)}{p\left({\overline{b}}_{i j}\Big|{T}_{i j}, c, k\right)}\cdot \frac{p\left({d}_{i j}\Big|\lambda, {p}_c, c, k\right)}{p\left({\overline{d}}_{i j}\Big|\lambda, {p}_c, c, k\right)}\right) $$

where *b*_*ij*_ and *T*_*ij*_ are B-allele read depth and total read depth of the *j*th heterozygous SNP in the *i*th segment, and *d*_*ij*_ is the corresponding read counts, ${\overline{b}}_{ij}$ and ${\overline{d}}_{ij}$ denotes the expected B-allele read depth and read counts associated with aberration state *c* and the *k*th clonal cluster, respectively. Furthermore, the scores for all segments are scaled to 0 ~ 100.

### Implementation of CLImAT-HET

An efficient implementation of CLImAT-HET by using C/Matlab is freely available at GitHub [27], our previously published tool DFExtract [19] is used to prepare input files for CLImAT-HET.

### Datasets

#### Real dataset

WGS data from 5 primary TNBC samples described in a previous study [28] are analyzed in this study. Each sample was sequenced at approximately 30× coverage on the Life/ABI SOLID sequencing platform.

#### Simulated dataset

To simulate tumors containing multiple clones, we first define one normal genome (denoted as *n*) and four tumor genomes (denoted as *a*, *b*, *c* and *d*), and then generate mixtures by mixing five genomes at predefined proportions. As illustrated in Figure S1 (Additional file 2), *a* is the main clone and the genome is generated by defining a number of segments along the reference, meanwhile each segment is specified by a copy number state including total copy number and major allele copy number. Genome *b* and *c* are constructed by introducing new aberrations into genome *a*, and genome *d* corresponds to the subclones deriving from the second clonal expansion based on *b*. We use a normal sample HCC1954-BL downloaded from CGHub [29] to generate sequencing data of the simulated genomes by following these steps: 1) For each segment of the genome, reads aligned to the region are randomly and repeatedly sampled from BAM file of the sample HCC1954-BL according to the copy number of the segment, 2) nucleotide sequences of the sampled reads are properly modified to match BAF values of the SNPs within each segment, and 3) the processed reads are merged to generate BAM files using SAMtools [30]. We generate mixtures of genomes by sampling reads from the BAM files at predefined proportions, and reads are sampled to 30× coverage at all mixtures. By this way, 20 simulated tumor samples (Table S2, Additional file 3) are generated to evaluate the performance of CLImAT-HET in detecting clonal and subclonal CNA and LOH segments.

For each simulated sample, the underlying cellularity of clonal clusters are computed based on the relationship between the tumor genomes making up the mixture. For example, the mixture “a_010b_030n_060” is made up by genomes *a*, *b* and *n* with respective proportions of p_a_ = 0.1, p_b_ = 0.3 and p_n_ = 0.6, thus there will be two clonal clusters with the underlying cellularity equal to 0.3 (p_b_) and 0.4 (p_a_ + p_b_); similarly, the mixture “a_020b_035c_025n_020” will yield three clonal clusters with cellularity of 0.35 (p_b_), 0.25 (p_c_) and 0.8 (p_a_ + p_b_ + p_c_), while the mixture “a_010b_010d_025n_055” contains three clonal clusters with cellularity of 0.25 (p_d_), 0.35 (p_b_ + p_d_) and 0.45 (p_a_ + p_b_ + p_d_).

### Competitive methods

Two advanced methods, OncoSNP-SEQ [23] and CLImAT [19], are adopted to make comparison between the performance of CNA and LOH detection algorithms. When running OncoSNP-SEQ on the simulated samples, the simulated SNP sites are used to prepare the input file during the preprocessing step, and we run the main program with the option “--chr_range [1:22], −-normalcontamination, −-maxnormalcontamination 0.8, −-seqtype illumina” on homogeneous samples, and with an additional option “--tumourheterogeniety” on heterogeneous samples. As OncoSNP-SEQ generally output multiple solutions per sample, and in this case we select the one associated with the maximum likelihood as the optimal solution. For CLImAT, we use the default configuration on all samples.

### Performance evaluation

For the simulated samples, copy number and genotype profiles of all segments predefined in simulation experiment are used as the golden standard for evaluation. Accuracy is calculated for each method by comparing the predictions with the ground truth. We consider a segment to be accurately identified only if any predicted segment covers the 75% size of the segment and have exact matching of total and major copy number with the segment. Accuracy is defined as the proportion of accurately identified segments among all segments.

## Results

In this section, we perform a comprehensive assessment of CLImAT-HET on both simulated and real datasets in terms of inferring cellularity of clonal clusters and identifying CNA/LOH segments.

### Results on simulated data

We employ a simulation study to evaluate the accuracy of our estimates of cellularity of clonal clusters, tumor purity and predictions of CNA/LOH segments. Our simulated data is generated based on a real normal sample HCC1954-BL downloaded from CGHub as described in the Methods. We run CLImAT-HET, OncoSNP-SEQ and CLImAT to infer CNA/LOH segments and associated cellularity.

**Fig. 2:**
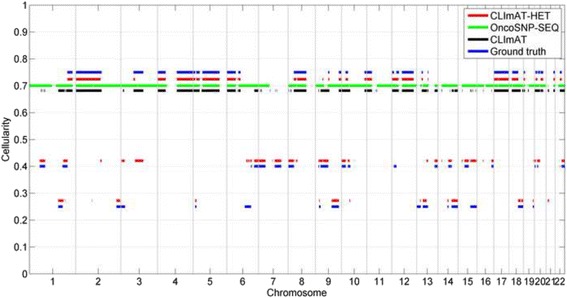
The cellularity estimation results of different methods on a simulated sample. Cellularity estimations of different methods on a simulated sample are compared with the ground truth. The predefined cellularity of all segments in simulation study are treated as underlying truth. CLImAT-HET correctly identify three clonal populations and infer their cellularity, meanwhile assign correct clonal cluster to 85% of all segments

**Fig. 3:**
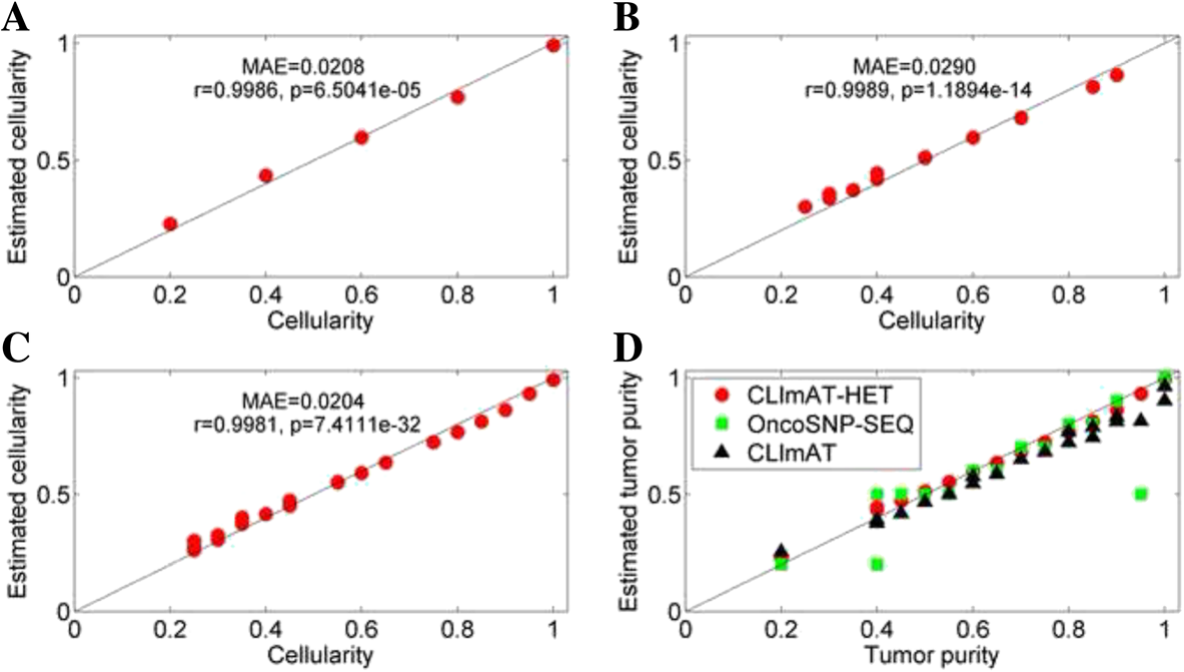
Cellularity prediction results of CLImAT-HET, and tumor purity estimation results of different methods on simulated samples. Estimated cellularity are compared with the underlying truth cellularity for each simulated sample. Results of CLImAT-HET on samples containing one (**a**), two (**b**) and three (**c**) clonal populations are shown respectively. Inferred tumor purities of each method are also compared with the ground truth tumor purities (**d**)

To assess the accuracy of cellularity estimations of CLImAT-HET, we compare the estimated cellularity to the ground truth cellularity for each clonal cluster. For each sample, the underlying cellularity of clonal clusters are computed based on the tumor genomes making up the mixture (more details in the Methods). CLImAT-HET is able to correctly infer the number of clonal clusters and corresponding cellularity for all the simulated mixtures. An example of cellularity estimations on a simulated sample “a_035b_015d_025n_025” with three clonal populations is shown in Fig. 2. CLImAT-HET correctly identify three clonal clusters and infer their cellularity (0.27, 0.41 and 0.72), simultaneously assign correct clonal cluster to 85% of all segments. OncoSNP-SEQ infers one clonal cluster with cellularity of 0.7, and CLImAT estimates the tumor purity as 0.68. Moreover, we compute the Pearson correlation coefficient (r) and mean absolute error (MAE) for cellularity estimations, and the results show that the estimated cellularity of CLImAT-HET has a highly significant positive correlation (r > 0.99, *p*-value < 5 × 10^−5^, MAE < = 0.02) with the underlying cellularity across all simulated samples (Fig. 3a, b and c), which demonstrates CLImAT-HET’s ability to precisely recover clonal architecture. We further assess the accuracy of tumor purity estimations using the same metrics, and strongly significant correlation (r = 0.997, *p*-value = 5.14 × 10^−22^, MAE = 0.023) for all the mixtures relative to the ground truth tumor purity is observed for CLImAT-HET (Fig. 3d). OncoSNP-SEQ accurately estimates the tumor purity for 90% of the samples (r = 0.878, *p*-value = 3.71 × 10^−7^, MAE = 0.053), and CLImAT also achieves good performance (r = 0.992, *p*-value = 9.75 × 10^−18^, MAE = 0.058).

**Fig. 4:**
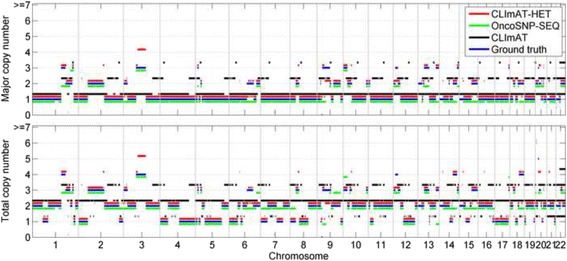
The copy number inference results of different methods on a simulated sample. Major and total copy numbers inferred by CLImAT-HET, OncoSNP-SEQ and CLImAT are compared with the ground truth, respectively. The predefined major and total copy numbers of all segments in simulation study are treated as underlying truth. The results show that CLImAT-HET infers the correct copy numbers for 86% of all segments

**Fig. 5:**
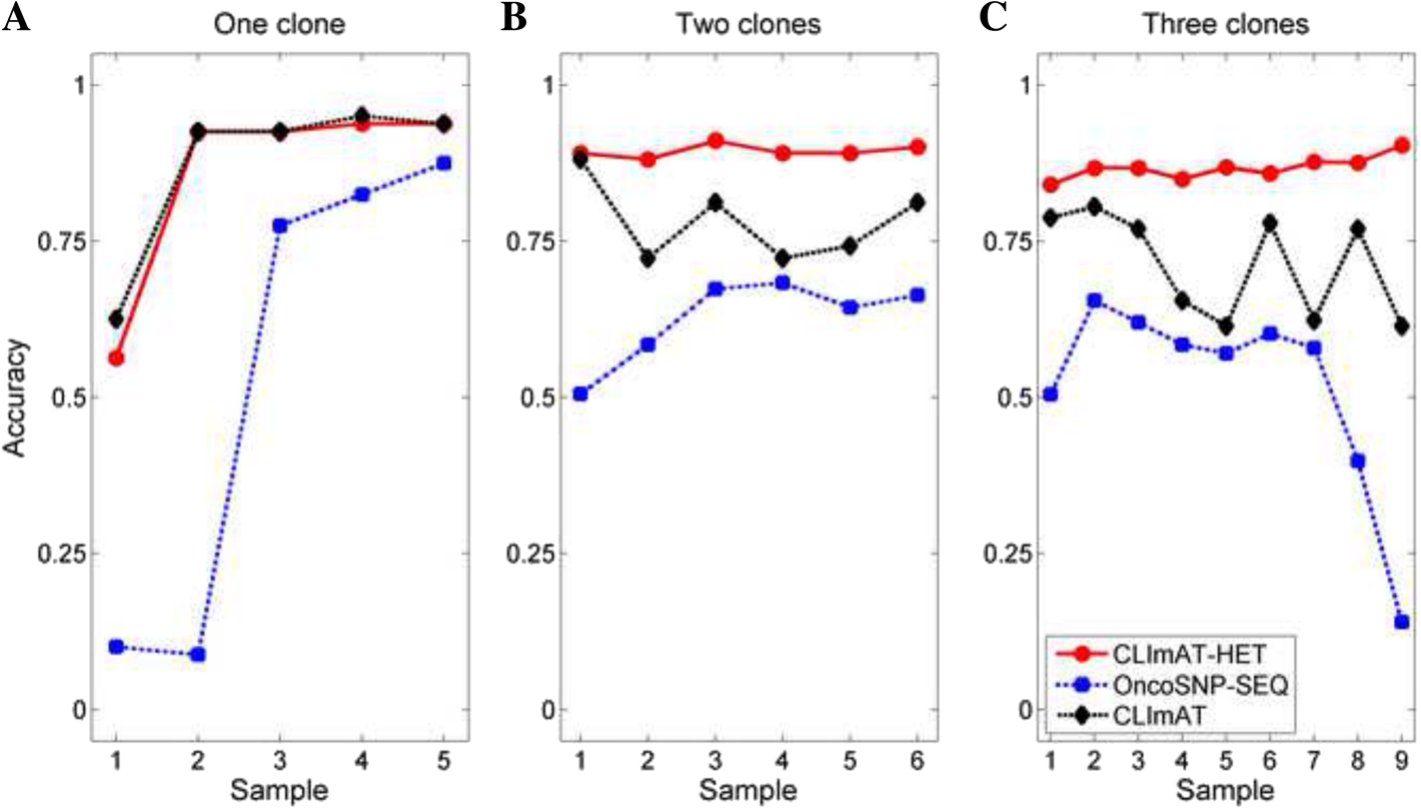
The accuracy of inferred copy numbers of different methods on simulated samples. The abilities of CLImAT-HET, OncoSNP-SEQ and CLImAT in inferring tumor genotypes are assessed in simulated dataset. A segment is considered to be accurately identified only if both the total and major copy numbers of the segment are accurately identified. For each simulated sample, the total and major copy number profiles of all segments predefined in simulation experiment are used as the golden standard for evaluation. The performance of different methods on homogeneous samples (**a**), tumor samples with two clonal populations (**b**) and three clonal populations (**c**) is assessed respectively, the x-axis represents the sample Id as defined in Additional file 3

Next, we proceed to assess the abilities of CLImAT-HET, OncoSNP-SEQ and CLImAT in inferring tumor genotypes. We consider a segment to be accurately identified if and only if both the total and major copy numbers are accurately identified, and the sizes of the segments are not considered. For each simulated sample, total and major copy number profiles of all segments predefined in simulation experiment are used as the golden standard for evaluation. CLImAT-HET is able to accurately infer the total and major copy numbers of each segment for all the simulated mixtures. An example result from the simulated sample “a_035b_015d_025n_025” is shown in Fig. 4. The results show that CLImAT-HET infers the correct tumor genotype for 86% of all segments, while OncoSNP-SEQ and CLImAT correctly identify the 61 and 68% of the segments, respectively. OncoSNP-SEQ presents a relatively lower performance because we adopt strict evaluation strategy. For a general evaluation, we use metric accuracy, which is defined as the proportion of accurately identified segments, to compare the performance of the different methods. The results on simulated data are shown in Fig. 5. For the homogeneous tumor samples (Fig. 5a), there is only one clonal cluster with cellularity equal to tumor purity, all methods show high accuracy when tumor purity is larger than 0.4. For the heterogeneous tumor samples (Fig. 5b and c), OncoSNP-SEQ and CLImAT achieve respective mean accuracies of 0.56 and 0.74, and CLImAT-HET accurately identify both the total and major copy numbers with a mean accuracy of 0.88. The performance of OncoSNP-SEQ is declining on some samples, possibly the reads coverage of these samples is not deep enough to enable OncoSNP-SEQ’s accurate inference of local parameters [23].

### Results on real data

We also examine CLImAT-HET on 5 primary triple negative breast cancer samples, which are sequenced at approximately 30× coverage and also assayed by Affymetrix SNP6.0 array [28]. ASCAT [31] is a widely used software to analyze SNP-array data, therefore we use it to infer the tumor purities.

**Fig. 6:**
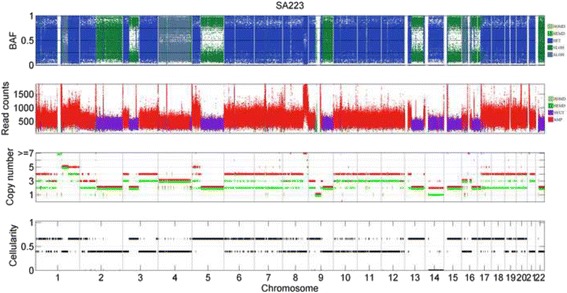
The subclonal prediction results of CLImAT-HET on sample SA223. CLImAT-HET infers sample SA223 as heterogeneous with two distinct clonal populations. The cellularity of one subclonal cluster is 0.66 and in good concordance with the tumor purities estimated by CLImAT, APOLLOH and ASCAT. In addition, CLImAT-HET identify another subclonal cluster with cellularity of 0.39

**Fig. 7:**
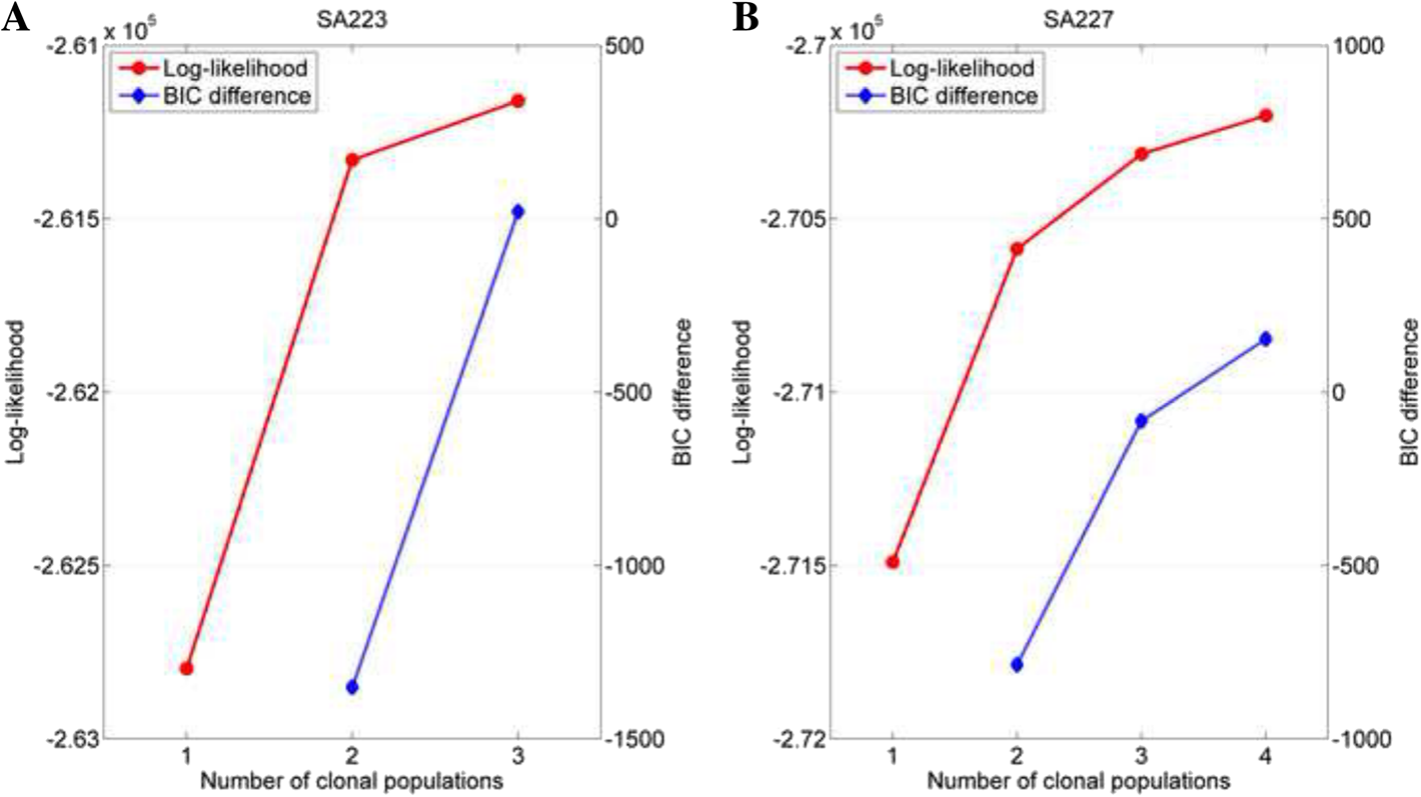
The log-likelihoods and BIC differences of sample SA223 and SA227 under different number of clonal populations. The log-likelihoods and BIC differences are measured under each iteration for sample SA223 (**a**) and SA227 (**b**). The iteration continues until the BIC difference is greater than zero. CLImAT-HET predicts the number of clonal populations as 2 and 3 for sample SA223 and SA227 respectively

The subclonal prediction results of CLImAT-HET on sample SA223 are shown in Fig. 6. CLImAT-HET identify one subclonal cluster with cellularity of 0.66, which is in good concordance with the tumor purities estimated by CLImAT, APOLLOH [13] and ASCAT (Table 1). This population presents copy neutral LOH spanning chromosomes 3p(22.3–11.1), 5q, 13q(13.3–33.2), 15q and 16q(13–24.3), and amplified heterozygous regions on chromosomes 1p, 1q(25.3–44), 5p, 6, 7, 9p, 17q, 18 and 20. ASCAT infers the measure of goodness of fit as 0.82 for sample SA223, showing that there may be aberration events represented in subclonal populations and not well interpreted by the models. Interestingly, CLImAT-HET identify another subclonal cluster with cellularity of 0.39, and copy neutral LOH regions on chromosomes 1q, 9q(21.2–34.3) and 22q, and segmental amplifications on chromosomes 3p(26.3–22.3), 3q(11.2–27.3), 4, 10, 11 and 12 are represented in the population. Figure 7a shows the log-likelihoods and BIC differences of sample SA223 under each iteration, and CLImAT-HET selects *K* = 2 as the optimal number of clonal populations since the BIC difference becomes to be positive when the number of clonal populations increases to 3.

**Table 1:** Tumor purity estimated by CLImAT, APOLLOH, CLImAT-HET and ASCAT for TNBC samples

Sample	CLImAT	APOLLOH^a^	CLImAT-HET	ASCAT
SA220	0.44	0.80	0.62	0.62
SA223	0.60	0.65	0.66	0.69
SA224	0.50	0.79	0.52	0.55
SA225	0.76	0.86	0.75	0.74
SA227	0.47	0.70	0.50	0.43

^a^The tumor purities estimated by APOLLOH are obtained from a previous study [13]

**Fig. 8:**
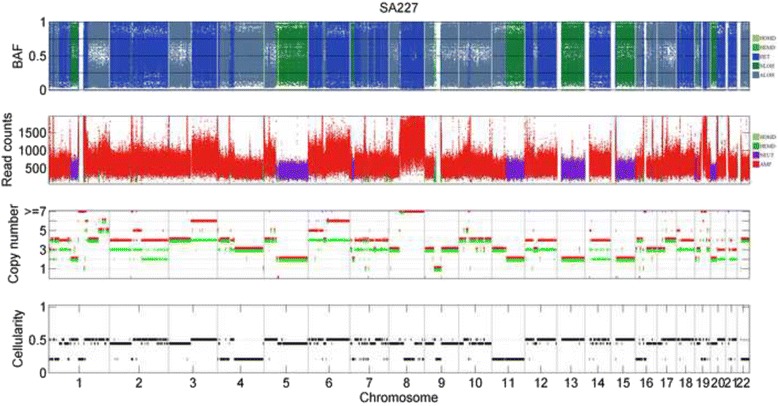
The subclonal prediction results of CLImAT-HET on sample SA227. CLImAT-HET infers sample SA227 as heterogeneous with three distinct clonal populations. The cellularity of one subclonal cluster is 0.44 and in accordance with the tumor purities estimated by CLImAT and ASCAT. In addition, CLImAT-HET identify other two subclonal clusters with respective cellularity of 0.5 and 0.21

For sample SA227, CLImAT-HET predicts it as heterogeneous with three distinct clonal populations, and the subclonal prediction results are shown in Fig. 8. One estimated subclonal cellularity of 0.44 is in accordance with the tumor purities estimated by CLImAT and ASCAT (Table 1). Copy neutral LOH on chromosome 5q and amplified LOH on chromosomes 3p, 8p and 9 are represented in this population. One other clonal cluster presents a similar cellularity of 0.5, which may correspond with the relatively higher measure of goodness of fit inferred as 0.9 by ASCAT. The genome of this clonal population is featured by a number of segmental amplifications across chromosomes 1, 2q, 3q, 5p, 6, 12, 17q, 18 and 20q, and copy neutral LOH regions spanning chromosomes 13 and 15. The third clonal cluster has a relatively lower cellularity of 0.21, and aberration events mainly include copy neutral LOH regions located at chromosome 11q and amplified LOH regions on chromosomes 4p, 11p, 16p and 22q. Figure 7b shows the log-likelihoods and BIC differences of sample SA227, and CLImAT-HET selects *K* = 3 as the optimal number of clonal populations.

For samples SA220, SA224 and SA225, the measures of goodness of fit returned by ASCAT are 0.95, 0.94 and 0.98 respectively, indicating these samples are probably homogeneous. Accordingly, CLImAT-HET analysis of these samples shows that there is also no significant evidence for existence of subclonal events, and the inferred cellularity of the single clonal population is 0.62, 0.52 and 0.75 respectively, which are in good concordance with the tumor purities predicted by CLImAT and ASCAT (Table 1). The copy number estimation results of these samples are shown in Figure S2-4 (Additional file 2).

## Discussion

CLImAT-HET is a novel statistical method for inferring subclonal CNA and LOH segments from WGS data of heterogeneous tumor samples. It is developed based on our previously published algorithm CLImAT, and take into account the issue of intra-tumor heterogeneity. Compared with CLImAT that only handles homogeneous tumor samples, CLImAT-HET can efficiently deal with both homogeneous and heterogeneous tumor samples. The read counts and read depths data generated from multiple cell populations is quantitatively represented, and proper emission models are adopted in the HMM. Furthermore, we integrate a BIC module into the CLImAT-HET framework to effectively determine the underlying number of subclonal populations. These features enable CLImAT-HET’s advantages in handling complex WGS data. First, the appropriate decomposition of mixed signals improves the CNA/LOH detection performance. Second, CLImAT-HET is more sensitive to the aberration events represented in minor cell populations when compared to existing methods as shown in simulated study. Third, CLImAT-HET is able to accurately infer the cellularity each subclonal cluster. Finally, the evaluation results on both simulated and real WGS data demonstrate the advantages of our algorithm.

CLImAT-HET also has limitations due to its adopted modeling assumptions. The assumption that only one aberrant genotype exists at each genomic locus will not hold if distinct subclonal populations have different aberrant genotypes at the same locus. However, it is difficult to distinguish among multiple subclones that have variable genotypes. The emission models of CLImAT-HET need to be extended to account for these situations, and the joint analysis of read counts and read depths signals may output multiple solutions. Identifying distinct haplotypes harboring linked mutations might be a good way to identify multiple mutations at the same locus in different populations.

## Conclusions

In summary, we demonstrate that CLImAT-HET represents remarkable advantages in inferring subclonal CNA/LOH segments from tumor WGS data. The high performance of CLImAT-HET benefits from delicate representation of copy number profiles of distinct cell populations, and efficient statistical methods for inference of the global parameters. We expect that CLImAT-HET will complement the arsenal of bioinformatics tools developed for investigating the role of tumor tumourigenesis and progression.

## Additional files

Additional file 1: Table S1. This file contains Supplementary Table S1, and provides the definition of copy number aberration states in CLImAT-HET. (PDF 104 kb)
Additional file 2: This file contains Supplementary Figures. (PDF 1702 kb)
Additional file 3: Table S2. This file contains Supplementary Table S2. (XLS 26 kb)

## Abbreviations

BAF: B allele frequency
BIC: Bayesian information criterion
CNA: Copy number alterations
EM: Expectation maximization
HMM: Hidden Markov model
LOH: Loss of heterozygosity
NB: Negative binomial
SNV: Single-nucleotide variants
SV: Structure variations
TNBC: Triple negative breast cancer
WGS: Whole-genome sequencing
