# Supplementary material for: CLImAT-HET: detecting subclonal copy number alterations and loss of heterozygosity in heterogeneous tumor samples from whole-genome sequencing data
Source: BMC Med Genomics. 2017 Mar 15;10:15. doi: 10.1186/s12920-017-0255-4 (PMC5351278; doi:10.1186/s12920-017-0255-4)
Supplement: Additional file 1: Table S1. — This file contains Supplementary Table S1, and provides the definition of copy number aberration states in CLImAT-HET. (PDF 104 kb) [file 12920_2017_255_MOESM1_ESM.pdf]

Table S1. Definition of copy number aberration states in CLImAT-HET

State	Copy number	(Tumor genotype, normal genotype)	Aberration status
1	0	(N/A, AA), (N/A, BB), (N/A, AB)	HOMD
2	1	(A,AA), (B,BB), (A,AB), (B,AB)	HEMD
3	2	(AA,AA), (BB,BB), (AB,AB)	NHET
4	2	(AA,AA), (AA,AB), (BB,BB), (BB,AB)	NLOH
5	3	(AAA,AA), (BBB,BB), (AAB,AB), (ABB,AB)	AHET
6	3	(AAA,AA), (AAA,AB), (BBB,BB), (BBB,AB)	ALOH
7	4	(AAAA,AA), (BBBB,BB), (AAAB,AB), (ABBB,AB)	AHET
8	4	(AAAA,AA), (BBBB,BB), (AABB,AB)	AHET
9	4	(AAAA,AA), (BBBB,BB), (AAAA,AB), (BBBB,AB)	ALOH
10	5	(AAAAA,AA), (BBBBB,BB), (AAAAB,AB), (ABBBB,AB)	AHET
11	5	(AAAAA,AA), (BBBBB,BB), (AAABB,AB), (AABBB,AB)	AHET
12	5	(AAAAA,AA), (BBBBB,BB), (AAAAA,AB), (BBBBB,AB)	ALOH
13	6	(AAAAAA,AA), (BBBBBB,BB), (AAAAAB,AB), (ABBBBB,AB)	AHET
14	6	(AAAAAA,AA), (BBBBBB,BB), (AAAABB,AB), (AABBBB,AB)	AHET
15	6	(AAAAAA,AA), (BBBBBB,BB), (AAABBB,AB)	AHET
16	6	(AAAAAA,AA), (BBBBBB,BB), (AAAAAA,AB), (BBBBBB,AB)	ALOH
17	7	(AAAAAAA,AA), (BBBBBBB,BB), (AAAAAAB,AB), (ABBBBBB,AB)	AHET
18	7	(AAAAAAA,AA), (BBBBBBB,BB), (AAAAABB,AB), (AABBBBB,AB)	AHET
19	7	(AAAAAAA,AA), (BBBBBBB,BB), (AAAABBB,AB), (AAABBBB,AB)	AHET
20	7	(AAAAAAA,AA), (BBBBBBB,BB), (AAAAAAA,AB), (BBBBBBB,AB)	ALOH
