# Supplementary material for: CLImAT-HET: detecting subclonal copy number alterations and loss of heterozygosity in heterogeneous tumor samples from whole-genome sequencing data
Source: BMC Med Genomics. 2017 Mar 15;10:15. doi: 10.1186/s12920-017-0255-4 (PMC5351278; doi:10.1186/s12920-017-0255-4)
Supplement: Additional file 2: — This file contains Supplementary Figures. (PDF 1702 kb) [file 12920_2017_255_MOESM2_ESM.pdf]

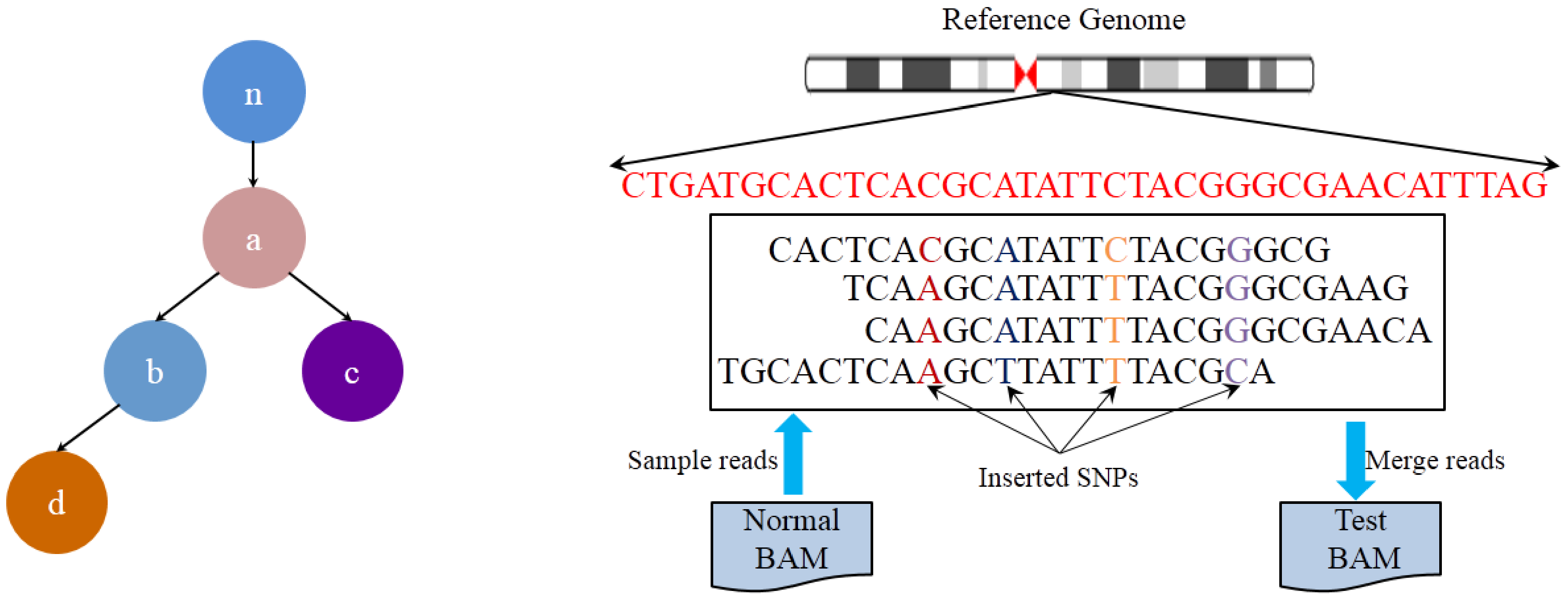

Figure S1. Simulation of tumor sample containing multiple subclonal populations. One normal genome and four tumor genomes are constructed. Population *a* is the main clone, population *b* and *c* are subclones derived from *a*, and population *d* corresponds to the subclones deriving from the second clonal expansion based on *b*. A real normal sample is used to generate sequencing data of the simulated genomes, reads are randomly sampled from the BAF file of the real normal sample and further processed to match the BAF of the inserted SNPs within each segment.

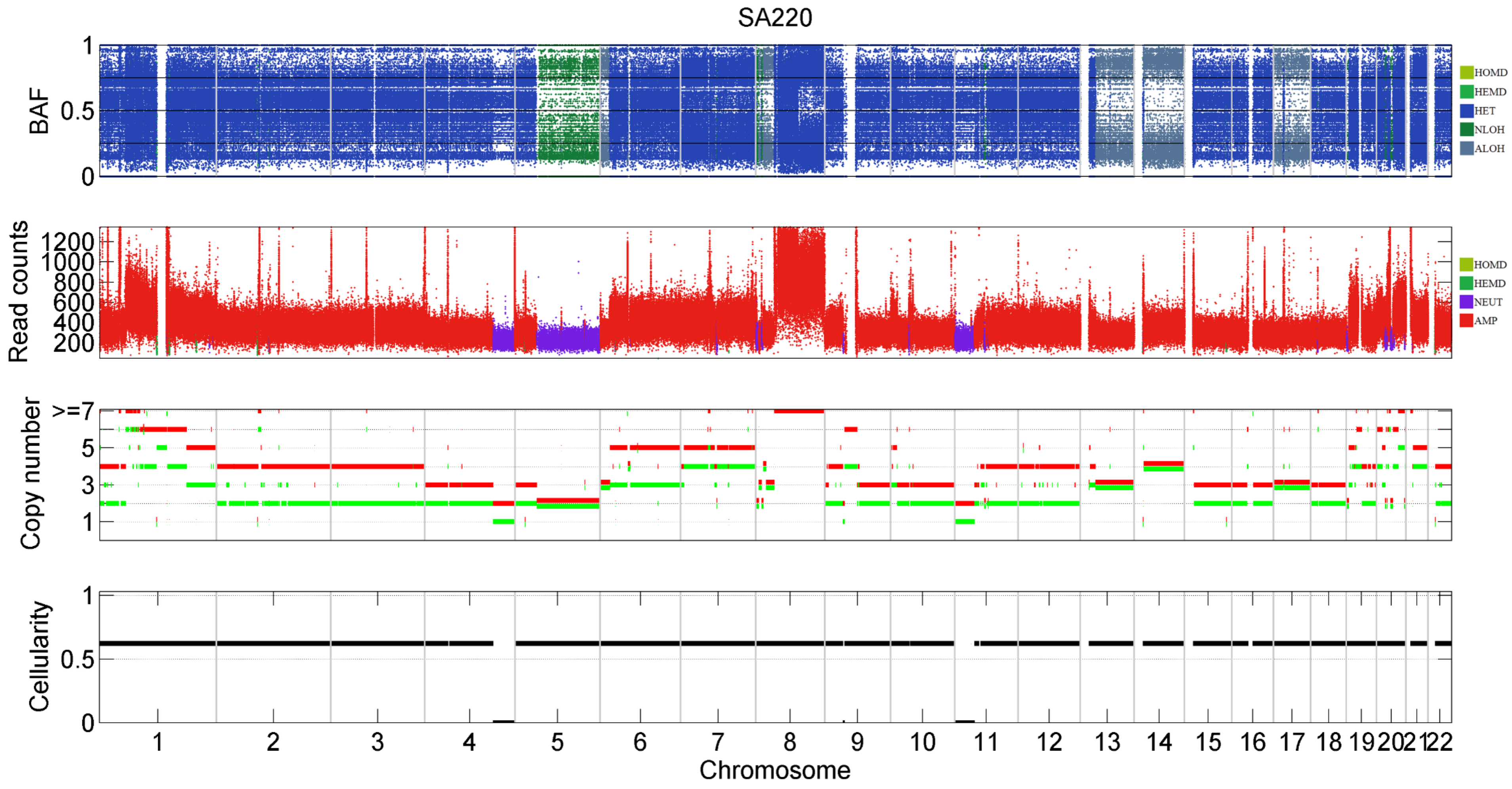

Figure S2. The subclonal prediction results of CLImAT-HET on sample SA220. CLImAT-HET predicts sample SA220 as homogeneous, the estimated tumor purity is 0.62.

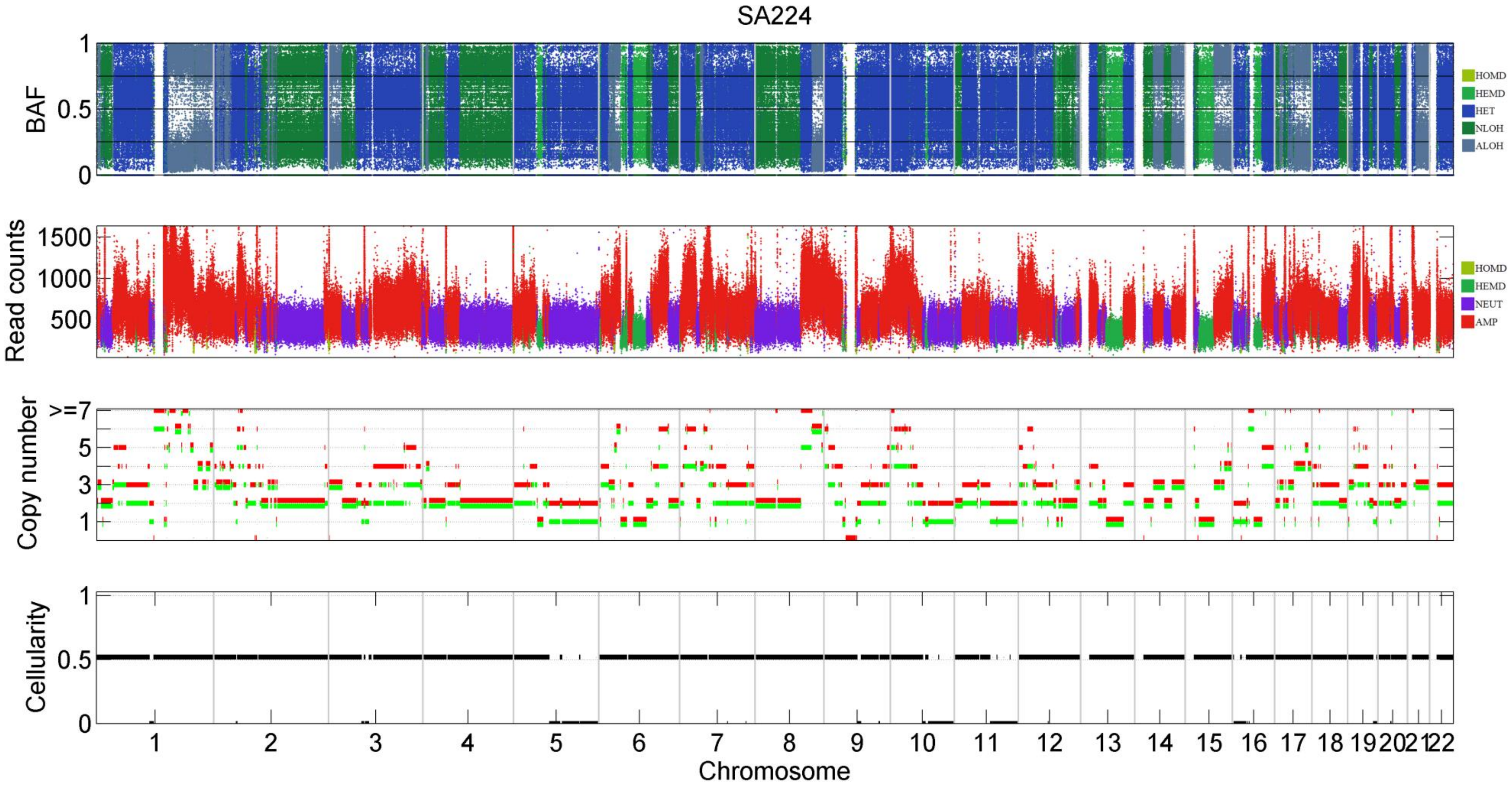

Figure S3. The subclonal prediction results of CLImAT-HET on sample SA224. CLImAT-HET predicts sample SA224 as homogeneous, the estimated tumor purity is 0.52.

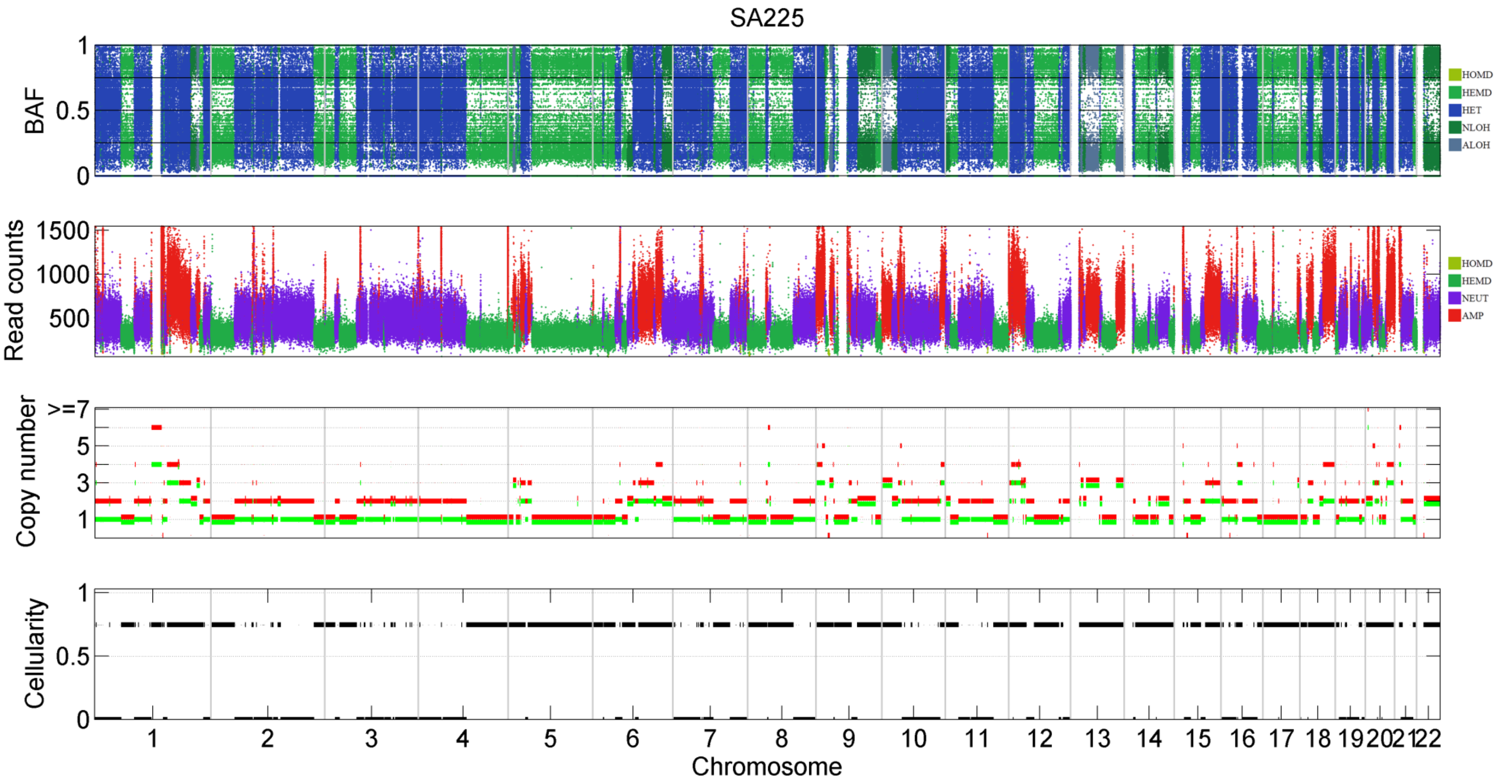

Figure S4. The subclonal prediction results of CLImAT-HET on sample SA225. CLImAT-HET predicts sample SA225 as homogeneous, the estimated tumor purity is 0.75.
